# Supplementary material for: Methamphetamine use disorder, perceived impacts, and associated factors among adults receiving care at Sri Lanka’s National Institute of Mental Health: An analytical cross-sectional study
Source: PLoS One. 2026 Jan 13;21(1):e0326469. doi: 10.1371/journal.pone.0326469 (PMC12798982; doi:10.1371/journal.pone.0326469)
Supplement: S1 Table — (DOCX) [file pone.0326469.s001.docx]

**Supplementary File S1**

**Thematic Codebook for Perceived Physical, Psychological, and Social Impacts of Methamphetamine Use (N = 427)**

**Coding Approach**

Open-ended responses to perceived impacts were analyzed using inductive thematic analysis. Codes were developed iteratively from the data, refined through consensus, and applied to all responses. Multiple codes were permitted per participant**.** Coding was conducted independently by two researchers, and discrepancies were resolved through discussion and consensus.

**Table 1. Physical Impact Codebook**

| **Code Name** | **Theme** | **Operational Definition** | **Inclusion Criteria** | **Exclusion Criteria** |
| --- | --- | --- | --- | --- |
| PHY1 | Weight loss | Self-reported unintentional reduction in body weight attributed to Methamphetamine use | Mentions of becoming thin, losing body mass, wasting | Weight change due to unrelated medical illness |
| PHY2 | Loss of appetite | Reduced desire to eat or skipping meals due to drug use | Poor appetite, “no hunger,” not eating | Fasting for religious or other reasons |
| PHY3 | Dental problems | Damage to teeth or oral cavity | Tooth decay, broken teeth, bleeding gums, “meth mouth” | Dental issues due to trauma or aging |
| PHY4 | Malaise | General feeling of weakness or discomfort | Fatigue, tiredness, weakness | Weakness due to acute infection |
| PHY5 | Chest pain | Pain or discomfort in chest region after drug use | Tightness, burning sensation in chest | Chest pain due to diagnosed heart disease |
| PHY6 | Cough | Persistent or frequent coughing | Dry cough, smoker’s cough after use | Cough due to respiratory infections |
| PHY7 | Dry mouth | Reduced salivation after using Methamphetamine | “Dry throat,” “sticky mouth” | Dehydration unrelated to drug use |
| PHY8 | Myalgia | Muscle pain or body aches | Muscle pain, body aches | Pain due to injury |
| PHY9 | Excessive sweating | Abnormally high sweating | Profuse sweating, night sweats | Sweating due to fever |
| PHY10 | Physical injuries | Injuries sustained while intoxicated | Cuts, bruises, wounds, fractures | Injuries unrelated to substance use |
| PHY11 | Headache | Recurrent or persistent headaches | Migraine, continuous headache after use | Pre-existing migraine disorder |
| PHY12 | Muscle cramps | Involuntary painful muscle contractions | Leg cramps, body stiffness | Electrolyte imbalance unrelated to drugs |
| PHY13 | Hair loss | Excessive hair shedding after prolonged use | Bald patches, thinning hair | Genetic alopecia |
| PHY14 | Jaw clenching | Repetitive clenching or grinding of teeth | Bruxism, jaw stiffness | Anxiety-related bruxism without drug link |
| PHY15 | Muscle rigidity | Extreme stiffness or immobility of muscles | Locked muscles, rigidity | Parkinsonian rigidity |
| PHY 16 | Dyspeptic symptoms | Upper gastrointestinal discomfort attributed to methamphetamine use, | Epigastric pain, burning sensation, bloating, nausea, or indigestion | Food related gastric pain |
| PHY 17 | Shortness of breathing | Subjective sensation of difficulty in breathing or breathlessness following methamphetamine use | Difficulty in breathing | Exercise or activity induced SOB |

**Table 2. Psychological / Mental Impact Codebook**

| **Code Name** | **Theme** | **Operational Definition** | **Inclusion Criteria** | **Exclusion Criteria** |
| --- | --- | --- | --- | --- |
| PSY1 | Irritability | Increased anger, frustration, or short temper | Feeling angry easily, verbal outbursts | Long-standing personality traits |
| PSY2 | Delusions | Fixed false beliefs | Paranoia, suspiciousness, false beliefs | Culturally accepted beliefs |
| PSY3 | Hallucinations | Sensory perceptions without external stimuli | Seeing or hearing things | Dreams, nightmares only |
| PSY4 | Sleep disturbances | Disruption in the normal sleep pattern | Insomnia, sleeplessness | Sleep changes due to shift work |
| PSY5 | Anxiety/fearfulness | Excessive worry, nervousness, panic | Constant fear, restlessness | Temporary fear due to life events |
| PSY6 | Feeling low | Persistent sadness, hopelessness | Crying spells, loss of happiness | Situational sadness after loss |
| PSY7 | Poor concentration | Reduced attention span or focus | Memory problems, confusion | Illiteracy-related learning difficulties |
| PSY8 | Suicidal ideation | Thoughts of self-harm or suicide | Desire to die, self-harm ideas | Past suicide attempt unrelated to current use |
| PSY9 | Homicidal ideation | Violent thoughts toward others | Desire to harm others | Anger without violent intent |
| PSY10 | Aggression | Physical or verbal violent behavior | Fighting, hitting, threats | Assertive behavior |
| PSY11 | Loss of interest | Lack of motivation or pleasure | No interest in work or family | Laziness |
| PSY12 | Restlessness/agitation | Inability to sit still | Pacing, agitation | Hyperactivity disorder |

**Table 3. Social Impact Codebook**

| **Code Name** | **Theme** | **Operational Definition** | **Inclusion Criteria** | **Exclusion Criteria** |
| --- | --- | --- | --- | --- |
| SOC1 | Interpersonal conflict | Conflicts with family, spouse, peers | Arguments, fights, separation | Minor disagreements |
| SOC2 | Financial problems | Economic hardship due to drug use | Debt, selling property, inability to meet expenses | Poverty unrelated to substance use |
| SOC3 | Stigmatization | Social labeling and discrimination | Being avoided, insulted, rejected | Self-imposed isolation |
| SOC4 | Social isolation | Withdrawal from social interaction | Avoiding people, staying alone | Religious solitude |
| SOC5 | Employment disruption | Job loss or poor work performance | Unemployment, absenteeism | Job loss due to unrelated downsizing |
| SOC6 | Legal problems | Conflict with law enforcement | Arrests, court cases | Traffic violations unrelated to drug |
| SOC7 | Poor role performance | Inability to fulfill social/family roles | Neglecting family duties | Temporary role failure |
| SOC8 | Academic difficulties | Poor educational performance | School dropout, failed exams | Academic failure due to illness |

## **Coding Rules that we followed**

- Each response could be assigned **multiple codes.**
- Coding was based on **explicit participant statements.**
- Vague expressions were coded only when a **clear drug-related attribution** was evident.
- All discrepancies were resolved through **consensus meetings.**
