# Supplementary material for: Methamphetamine use disorder, perceived impacts, and associated factors among adults receiving care at Sri Lanka’s National Institute of Mental Health: An analytical cross-sectional study
Source: PLoS One. 2026 Jan 13;21(1):e0326469. doi: 10.1371/journal.pone.0326469 (PMC12798982; doi:10.1371/journal.pone.0326469)
Supplement: S2 Table — (DOCX) [file pone.0326469.s002.docx]

Supplementary File S2

**Exemplar Participant Quotes by Theme**

This supplementary file presents anonymized representative chosen participant quotations illustrating each major theme identified through thematic analysis.

**Table 1. Physical Impacts – Exemplar Quotes**

| **Theme** | **Exemplar Quote** |
| --- | --- |
| Weight loss | “I became very thin and weak after using ICE regularly.” |
| Loss of appetite | “I can stay the whole day without eating after taking the drug.” |
| Dental problems | “My teeth started breaking and bleeding after long-term use.” |
| Malaise | “Even small work makes me extremely tired now.” |
| Chest pain | “Sometimes my chest burns badly after using ICE.” |
| Cough | “I keep coughing continuously after smoking it.” |
| Dry mouth | “My mouth becomes very dry and sticky for hours.” |
| Myalgia | “There is a strong pain in my body after using.” |
| Excessive sweating | “I sweat heavily even when I am just sitting.” |
| Physical injuries | “I fell and got injured when I was under the influence.” |
| Headache | “I always get severe headaches after using ICE.” |
| Muscle cramps | “Sudden cramps start in my legs at night.” |
| Hair loss | “My hair started falling rapidly after continuous use.” |
| Jaw clenching | “My jaw tightens and I grind my teeth without control.” |
| Dyspeptic symptoms | “After taking the drug, I always get burning pain in my stomach and feel like vomiting.” |
| Shortness of breathing | When I use ICE, I feel tightness in my chest and I struggle to breathe |
| Muscle rigidity | “Sometimes my whole body becomes stiff and immobile.” |

**Table 2. Psychological Impacts – Exemplar Quotes**

| **Theme** | **Exemplar Quote** |
| --- | --- |
| Irritability | “I get angry even for very small things now.” |
| Delusions | “I always feel someone is following me.” |
| Hallucinations | “I hear voices when nobody is around.” |
| Sleep disturbances | “I cannot sleep for two or three days after using.” |
| Anxiety and fearfulness | “I feel very scared without any reason.” |
| Depression | “I feel hopeless and tired of life.” |
| Poor concentration and attention | “I cannot concentrate on anything for long.” |
| Suicidal and self-harm | “Sometimes I feel it is better to die than live.” |
| Homicidal ideation | “I suddenly feel like hurting people.” |
| Aggression | “I have physically attacked people when intoxicated.” |
| Loss of interest | “Even my children do not interest me anymore.” |
| Feeling low | When the drug effect goes away, I feel very low, hopeless, and useless. |
| Homicidal ideas | When I take the drug, I suddenly feel like attacking or killing people without reason |
| Restlessness and agitation | “I keep walking around without being able to sit.” |

**Table 3. Social Impacts – Exemplar Quotes**

| **Theme** | **Exemplar Quote** |
| --- | --- |
| Interpersonal conflict | “My constant fighting destroyed my family life.” |
| Financial problems | “I sold my household items to buy the drug.” |
| Stigmatization and social isolation | “People in my village avoid me now.” |
| Social isolation | “I prefer staying alone all the time.” |
| Employment disruption | “I lost my job because I was absent repeatedly.” |
| Legal problems | “I was arrested twice for drug-related charges.” |
| Poor role performance | “I failed as a father because of my addiction.” |
| Academic difficulties | “I dropped out of school because of drugs.” |
