# Supplementary material for: Methamphetamine use disorder, perceived impacts, and associated factors among adults receiving care at Sri Lanka’s National Institute of Mental Health: An analytical cross-sectional study
Source: PLoS One. 2026 Jan 13;21(1):e0326469. doi: 10.1371/journal.pone.0326469 (PMC12798982; doi:10.1371/journal.pone.0326469)
Supplement: Attachment — Submitted filename: Response to Reviewer.pdf [file pone.0326469.s003.pdf]

Dr. K.A.Sriyani,

Corresponding author.

Dear editor-in-chief and reviewers,

PLOS ONE,

We would like to sincerely thank you for the time and effort you have invested in reviewing our manuscript titled “Methamphetamine addiction and its perceived impact on adult clients at the National Institute of Mental Health, Sri Lanka: A descriptive cross-sectional study”. We appreciate the constructive feedback provided by the reviewers and editor, which has helped us improve the quality and clarity of our work.

Below, we provide detailed responses to each of the comments raised. All revisions have been made in the manuscript, with changes denoted as track changes (Both clean and track changed versions uploaded). We have addressed each point as follows:

Number	Reviewer’s Comment	Authors response
**Journal requirements**		
01.	Please ensure that your manuscript meets PLOS ONE's style requirements, including those for file naming.	The manuscript is rearranged to meet PLOS ONE requirements.
02.	Please describe in your methods section how the capacity to provide consent was determined for the participants in this study. Please also state whether your ethics committee or IRB approved this consent procedure. If you did not assess capacity to consent please briefly outline why this was not necessary in this case.	As recommended, we have revised the **Ethical Considerations** section of the manuscript to clarify that while a formal capacity assessment was not conducted, this was not considered mandatory in our study context. Only participants who were free from cognitive impairment, clinically stable, and deemed mentally competent by the treating mental health professionals were included. The consent procedure, including this inclusion criterion, was approved by the Ethics Review Committee of the National Institute of Mental Health (ERC No: 205/03/2023).
03.	We note that you have indicated that there are restrictions to data sharing for this study. For studies involving human research participant data or other sensitive data, we encourage authors to	All raw data is available at public repository. The DOI is stated within the submission system,<br><br>10.6084/m9.figshare.29816636

	share de-identified or anonymized data.<br>Please update your Data Availability statement in the submission form accordingly.	
**Reviewer 01**		
01.	Although the authors indicate that the study site admits patients from allover the country, admissions are basically from the Western province and this limits the applicability of data to other regions of the country. The sample is highly selective.	Thank you very much for your consideration. The study purposively included all eligible adult clients diagnosed with methamphetamine use disorder admitted to NIMH during the study period, irrespective of their district of origin, ensuring clinical consistency. Nonetheless, since the majority of admissions to NIMH were from the Western Province, the sample may not fully represent the geographic diversity of methamphetamine users across Sri Lanka, and it was stated under the limitations of the study.
02.	The described physical and mental impacts cannot be attributed to methamphetamine, as most of them were due to other psychoactive drugs or agents.	We appreciate the reviewer's insightful observation regarding the potential confounding effect of polydrug use in attributing specific impacts to methamphetamine.<br><br>We agree that in a clinical sample where polydrug use is common, exclusive attribution of observed impacts to methamphetamine is scientifically challenging. However, we would like to clarify that our primary intention was to explore self-reported and perceived impacts associated with methamphetamine use among clients diagnosed with methamphetamine use disorder, rather than to establish causality.<br><br>Given that methamphetamine was the primary substance of use and the basis for admission in these clients, their perceived experiences remain highly relevant for clinical management and health education, despite potential contributions from other substances.<br><br>Additionally, in real-world clinical and harm reduction contexts, it is often difficult to isolate the impacts of individual substances due to the high prevalence of concurrent substance use.

		We have revised the **title, objectives, and discussion** to explicitly reflect that our study focuses on **"Perceived Impacts of Methamphetamine Use"** rather than definitive impacts attributable solely to methamphetamine. We believe this adjustment maintains the value of the study while addressing the reviewer's valid concern about attribution.
03.	The physical effects described are very nonspecific. e.g weight loss (subjective or objective), cough (was it based on the duration or severity), anorexia	We acknowledge that physical impacts such as weight loss, cough, and anorexia are nonspecific and can have multiple causes. However, in this study, data were collected through client self-reports during data collection, aiming to capture the perceived impacts experienced by clients diagnosed with methamphetamine use disorder during their use period, and this matter was clearly explained during the information-giving period for the patient. The intention was not to present these as clinical diagnostic findings but to document clients' lived experiences, which are valuable for understanding patient perspectives in treatment and education planning. We have clarified this in the manuscript to reflect that the findings represent perceived, self-reported impacts, not objectively measured clinical outcomes.
**Reviewer 02**		
	**Introduction**	
1.	The opening sentence is acceptable but could be made more impactful by highlighting why this stimulant poses a particular threat in low- and middle-income country contexts, where treatment and harm-reduction services are limited.	Accepted the comment, and the information was added at the end of the opening sentence.
2.	The transition from global to Asian data is abrupt. Adding a bridging sentence would improve the flow (e.g., "This global trend is reflected in Asia, where…").	The transition was smoothed by adding a bridging sentence, as the reviewer mentioned.

3.	Ensure numerical comparisons are clear and impactful; for example, show percentage increases when comparing 2022 to 2023 arrest data.	The numerical comparisons were re-reported in an impactful manner.
4.	Some phrasings are redundant (e.g., “widespread effects that are multifaceted and interrelated…” could be simplified).	The phrase “widespread effects that are multifaceted and interrelated” has been streamlined to “affects individuals across physical, psychological, and social domains,” which is more concise yet retains the full meaning. Other slight adjustments enhance flow and eliminate minor redundancy (e.g., avoiding unnecessary repetition of "users also reported...")
5.	When citing multiple sources, consider combining them for readability.	The changes are made in identified areas.
6.	The rationale for the study could be clearer: specify how this study will explore local motivations in depth.	The rationale was clearly and comprehensively mentioned, which emphasizes the lack of community-based, qualitative research.
7.	The research gap paragraph should be more assertive: e.g., “Despite rising prevalence, there is a critical lack of detailed, community-based research…”	The research gap was rephrased more assertively.
8.	Split the aim statement into clear, bullet-pointed objectives for easy reading.	As the reviewers commented, the aim statement was split and bullet-pointed separately.
9.	Minor grammatical edits: use “aged 12 or older” instead of “age 12 or older”; merge repetitive sentences for conciseness.	The grammatical mistake was corrected as aged 12 or more.
	**Methods**	
10.	Important details on sampling and recruitment procedures are missing. How were participants identified and selected?	Detailed how participants were identified and selected.
11.	Use consistent past tense for the study design description. Avoid repetition when describing the setting.	The description of the setting was paraphrased, and repetitions were removed, and the tense was corrected.
12.	Clarify how DSM-5 diagnoses were confirmed — by whom and based on what process?	Clarified who confirmed DSM-5 diagnoses and how.

		“”as confirmed by consultant psychiatrists or trained psychiatric medical officers using structured clinical interviews and medical records”
13.	Explain how withdrawal symptoms were identified and excluded during recruitment.	Withdrawal symptoms were identified through medical records and clinical observation by attending clinicians before recruitment.
14.	Confirm that the source for the DSM-5 criteria is cited correctly.	Re-checked the citation.<br>“American Psychiatric Association. Diagnostic and Statistical Manual of Mental Disorders. American Psychiatric Association; 2013. “
15.	For the pattern-of-use questions, indicate whether these items were validated or developed by the authors. If the author developed, how were they pre-tested and improved?	This one is authored and developed. At the end of the data collection tool, all validation methods, including content validity and pretest, were already described.
16.	You mention open-ended questions but do not explain how responses were coded and analyzed — this should be described briefly.	Described at the end of the data analysis
17.	Good to note the pre-test and Cronbach’s alpha; specify how feedback from the pre-test informed final revisions	We have updated the methodology section to clarify that participant feedback during the pre-test focused on item clarity and wording. Based on this input, minor revisions were made to simplify the language and improve understanding of key items, particularly those related to the frequency of use and psychological effects. Cronbach's alpha for 11 11-item scale is already mentioned.
18.	In data collection, mention how many interviewers were involved and whether inter-rater reliability was checked	It was already mentioned as “Data were collected by four investigators who had undergone training,…….”<br><br>It was measured by kappa values
19.	In data analysis, clarify that only descriptive statistics were planned if no inferential analysis was done; explain how missing data were handled.	No missing data were observed within the data sheet
20.	Ensure consistent tense and phrasing: e.g., “due to the population represents…” should be “because the population represents…”	This wording is corrected.

21.	it is not clear why the data was only analyzed descriptively. Rigorous data analysis would benefit the study. what confounding factors were detected, and how were they controlled?	Thank you for this important observation. This study was intentionally designed as a descriptive cross-sectional study with the primary aim of exploring the patterns, severity, and perceived impacts of methamphetamine addiction among a clinical population. Since there is a paucity of baseline data in this context, particularly regarding MA addiction and its psychosocial correlates, the study sought to first generate foundational insights rather than test specific hypotheses.<br><br>Given that the study focused on perceived impact rather than causal inference, and that variables such as addiction severity were not manipulated or independently controlled, adjustment for confounding was not applicable within this design. Moreover, many variables were exploratory and based on patient perception, making them less suitable for inferential analysis without validated outcome measures.<br><br>We fully agree that future research should incorporate analytical designs such as cohort studies or regression-based modeling to identify predictors, control confounding factors, and determine statistically significant relationships.<br><br>This study will be a baseline study for future research.
	**Results**	
22.	It is unclear why “Muslim” is classified as an ethnicity rather than a religious group — please clarify or adjust the categorization.	In this study, the term Muslim is changed to “Moor” to refer to the ethnic group commonly known as Sri Lankan Moors, who are predominantly Muslim by religion. While “Muslim” is often used interchangeably in local discourse, it primarily denotes religious affiliation. To avoid conflation of ethnicity and religion, and in alignment with national census classifications, the term “Moor” has been adopted to represent this ethnic group.
23.	The education level categories (primary, secondary, tertiary,	In Sri Lanka, the education level is categorized as primary education (grades 1 to 5),

	higher, vocational) are not clearly defined — consider aligning with standard classifications.	secondary education (grades 6 to Advanced level), tertiary (concurrently used as higher) education (undergraduate and/or postgraduate), and vocational and technical education.<br><br>The necessary changes were made in the table, and put a foot note to the table
24.	Use the consistent past tense when reporting results.	The consistency was maintained throughout the results section.
25.	Ensure small percentages are formatted consistently.	All percentage was rounded up to two decimal points.
26.	Tables are generally well-structured, but check for decimal alignment. Clarify in the polydrug use table that multiple responses were possible.	Decimal correction was made in the tables.<br><br>We have clarified in the text and that multiple responses were possible among polydrug users. This ensures that the reader understands the percentages may exceed 100% due to overlapping substance use.
27.	Heatmaps should be correctly labeled in the text as “Figure 1A,” “Figure 1B,” etc., and figures should be cross-referenced appropriately.	The figures are cross-referred as advised.
28.	When reporting severity and patterns, add a line to interpret what the distribution implies.	A new sentence was added to imply the distribution overall.
29.	Small details: use “Separated” instead of “Separate” for social status.	Correct the word as separated.
30.	Clarify the overlap between similar items such as “isolation from family” and “lack of family closeness.”	Thank you for your observation. While these items may appear similar, they address distinct aspects of familial relationships. *“Isolation from family”* refers to the behavioral or physical separation from family members, such as not living with them, avoiding contact, or being excluded from family activities. In contrast, *“lack of family closeness”* captures the emotional dimension specifically, the absence of warmth, trust, or supportive bonds even when regular contact exists. To clarify the conceptual distinction between these two items, we have added a footnote below the relevant table.

31.	Briefly interpret the significance of polydrug use and social impacts to guide the reader's understanding.	A brief sentence is added to emphasize the significance of this finding under the results section. In the discussion, this matter was discussed.
	**Discussion**	
32.	The Discussion should more clearly compare the study's findings with previous research, highlighting reasons for similarities and differences.	While appreciating the reviewer's insightful suggestion, we acknowledge the importance of situating our findings within the context of existing literature. However, as methamphetamine (MA) addiction, particularly in the Sri Lankan context, has received limited scholarly attention, there was a scarcity of directly comparable studies. Despite this limitation, we made every effort to integrate and critically engage with the available national and international literature relevant to substance use patterns, influence, and associated impacts. Where direct comparisons were not possible due to contextual or thematic gaps in prior research, we highlighted the novelty and significance of our findings and their implications for future studies and interventions.
33.	Some points are repeated unnecessarily (e.g., male predominance, cultural stigma, physical impacts). Combine these for conciseness.	Upon careful review of the manuscript, we found that while some related ideas were mentioned in multiple sections for thematic clarity, unnecessary repetition was minimal. However, to improve conciseness and flow, we have made targeted edits to streamline overlapping content
34.	Expand on the implications of polydrug use for treatment planning, withdrawal management, and relapse prevention in local contexts.	The implications of polydrug use are expanded for clear understanding.
35.	For physical impacts, tie findings to specific clinical consequences (e.g., the need for nutritional or dental interventions).	Necessary changes are made aligns with the comment.
36.	When citing other studies, integrate citations meaningfully: show how your results align with or differ from theirs.	As methamphetamine addiction, particularly in the Sri Lankan and Asian context, remains a significantly under-researched area, we encountered a scarcity of directly comparable studies that align closely with our findings. As such, in many instances, it was challenging to

		draw clear cut comparisons between our results and existing literature. However, where relevant data were available we have revised the discussion to integrate those citations more meaningfully by explicitly noting similarities or distinctions. These revisions aim to enhance the contextual relevance and interpretive clarity of our findings within the broader evidence base.
37.	Limitations are acknowledged but could be stronger: note the reliance on self-reported data, the potential for under- or over-reporting, and the lack of generalizability due to the single-site setting.	Limitation section is revised as mentioned in the comment.
38.	Highlight the strengths of the study (e.g., high response rate, comprehensive coverage of impacts).	The strengths are embedded, and the limitation section is renamed as Strengths and Limitations
39.	Offer recommendations for future research, such as using mixed methods or longitudinal designs.	Recommendations are provided as commented.
	Conclusion	
40.	The conclusion should be more precise and actionable. Avoid generic phrases like “sheds light”; instead, state what this study adds empirically and how it informs policy and practice.	The conclusion was revised to align with the comments.
41.	Summarize clear, evidence-based recommendations: e.g., the need for peer-led prevention, youth-focused outreach, integrated mental health services, and stricter control of Methamphetamine supply chains.	Add clear evidence-based recommendations.
