# Supplementary material for: Methamphetamine use disorder, perceived impacts, and associated factors among adults receiving care at Sri Lanka’s National Institute of Mental Health: An analytical cross-sectional study
Source: PLoS One. 2026 Jan 13;21(1):e0326469. doi: 10.1371/journal.pone.0326469 (PMC12798982; doi:10.1371/journal.pone.0326469)
Supplement: Attachment — Submitted filename: Table of comment.docx [file pone.0326469.s004.docx]

Dr. K.A.Sriyani,

Corresponding author.

Dear editor-in-chief and reviewers,

PLOS ONE,

We would like to sincerely thank you for the time and effort you have invested in reviewing our manuscript titled “Methamphetamine addiction and its perceived impact on adult clients at the National Institute of Mental Health, Sri Lanka: A descriptive cross-sectional study”. We appreciate the constructive feedback provided by the reviewers and editor, which has helped us improve the quality and clarity of our work.

Below, we provide detailed responses to each of the comments raised. All revisions have been made in the manuscript, with changes denoted as track changes (Both clean and track-changed versions uploaded). We have addressed each point as follows:

**Reviewer comments**

| **Number** | **Reviewer’s Comment** | **Authors response** |
| --- | --- | --- |
| 1. | How were open-ended responses systematically coded (beyond thematic grouping)? | Open-ended responses were systematically analyzed using an inductive thematic coding approach. Initial codes were generated directly from the data and refined through iterative reading. Two independent researchers applied the codes, and any discrepancies were resolved through discussion, with a third researcher providing arbitration when required. The finalized codes were then organized hierarchically into broader themes and categories, which allowed for systematic identification of patterns across responses beyond simple grouping. |
| 2. | Was the DSM-5 checklist interviewer-administered or self-reported? | DSM-5 assessment was the 2^nd^ section of the questionnaire. Initially, we mentioned that the whole questionnaire was interviewer-administered. Therefore, the DSM-5 checklist is interviewer-administered. |
| 3. | The reported Cronbach’s alpha (0.89) applies only to severity items, not the entire instrument. The reliability and validity of other sections remain unclear. | We appreciate the reviewer’s comment regarding the reliability analysis of the 10-item scale assessing reasons for MA use. Cronbach’s alpha was calculated, but the result was negative. This occurs because Cronbach’s alpha assumes that all items measure a single underlying construct (unidimensionality) and are positively correlated [1]. In our questionnaire, the 10 items represent distinct reasons for MA use, which are conceptually different and not necessarily expected to correlate positively. Therefore, Cronbach’s alpha is not an appropriate measure of internal consistency for this type of checklist.  Instead of relying on alpha, we examined the face and content validity of the items, ensuring each item captures a unique and relevant reason for MA use, and used descriptive statistics to report their prevalence. This approach is consistent with prior studies using similar “reason checklists” in substance use research.  [1] |
| 4. | Addiction severity is classified according to DSM-5 symptom count, but it is unclear whether clinicians verified responses or whether lay interviewers applied criteria. Misclassification risk should be acknowledged. | We thank the reviewer for the comment regarding the potential for misclassification. In our study, all participants were clinically diagnosed with methamphetamine use disorder according to DSM-5 criteria by consultant psychiatrists or psychiatric medical officers using structured clinical interviews and medical records. Therefore, the risk of misclassification is minimized as diagnoses were clinician-verified rather than relying solely on self-report. |
| 5. | The analysis is largely descriptive. Inferential or multivariate analyses (e.g., associations between demographics and severity) could provide greater depth. | Associated factors were calculated using chi-square and likelihood ratios. Findings were discussed in the discussion. |
| 6. | No adjustment for multiple testing is reported, despite numerous outcome measures. This raises concern about inflated type I error. | A clarification has been added in the “Data Analysis” section explaining that the study’s descriptive intent did not require adjustment for multiple comparisons, and results were interpreted accordingly [2] |
| 7. | The manuscript often implies causality (e.g., “methamphetamine causes family separation”) despite the cross-sectional design. The authors should temper causal language. | Accordingly, we carefully reviewed the entire Discussion section and rephrased all statements that implied causation to reflect associations instead. |
| 8. | The very high rate of polydrug use (87%) is striking but insufficiently discussed. How does this affect the interpretation of methamphetamine-specific impacts? Many “perceived impacts” may be attributable to multiple substances. | We have expanded the discussion to address how the high prevalence of polydrug use complicates the interpretation of methamphetamine-specific effects. A new paragraph has been added (highlighted in the revised manuscript) explaining the potential confounding influence of concurrent substance use and the need for future studies to isolate methamphetamine-related outcomes |
| 9. | Strengths and limitations should be presented more systematically. Currently, limitations are underplayed. | We revised the “Strengths and Limitations” section to systematically acknowledge study design, sampling, and measurement limitations, emphasizing their implications for interpretation. |
| 10. | Several grammatical and typographical errors reduce clarity (e.g., “boos energy” → “boost energy”; “ticket the box” → “ticked the box”). | All are corrected as necessary. |
| 11. | Figures/heatmaps are visually appealing but need clearer legends and consistency in labeling. | The figure reporting consistency was rearranged, and the legends now clearly illustrating the meaning of the figure. |
| 12. | Conclusions should align more closely with the descriptive nature of the study and avoid policy overreach without stronger evidence. | The conclusion has been rewritten to reflect the descriptive nature of the study and to avoid policy overreach, focusing instead on evidence-informed implications. |

References

1.	Tavakol M, Dennick R. Making sense of Cronbach’s alpha. Int J Med Educ. 2011 Jun 27;2:53–5.

2.	Rothman KJ. No adjustments are needed for multiple comparisons. Epidemiology. 1990 Jan;1(1):43–6.
