# Supplementary material for: Methamphetamine use disorder, perceived impacts, and associated factors among adults receiving care at Sri Lanka’s National Institute of Mental Health: An analytical cross-sectional study
Source: PLoS One. 2026 Jan 13;21(1):e0326469. doi: 10.1371/journal.pone.0326469 (PMC12798982; doi:10.1371/journal.pone.0326469)
Supplement: Attachment — Submitted filename: Response letter- Added.docx [file pone.0326469.s005.docx]

Dear Editor-in-Chief and all reviewers,

We would like to sincerely thank you for the time and effort you have invested in reviewing our manuscript titled “Methamphetamine addiction and its perceived impact on adult clients at the National Institute of Mental Health, Sri Lanka: A descriptive cross-sectional study”. We appreciate the constructive feedback provided by the reviewers, which has helped us improve the quality and clarity of our work.

Below, we provide detailed responses to each of the comments raised. All revisions have been made in the manuscript, with changes denoted as track changes (Both clean and track-changed versions uploaded). We have addressed each point as follows

Thank you

| Reviewer’s comment | Authors response | Remarks |
| --- | --- | --- |
| The paper repeatedly infers or implies determinants of severity from cross-sectional and largely descriptive/bivariate data. Causality cannot be inferred. Please re-frame throughout as associations among treated in-patients/outpatients at NIMH. | Thank you for this important observation. We acknowledge that our cross-sectional design and descriptive/bivariate analyses do not permit causal inferences. We have revised the manuscript to remove any causal language and now describe the findings strictly as associations among treatment-seeking in-patients and out-patients at NIMH. All relevant sections have been updated accordingly. |  |
| Diagnostic ascertainment is described as “DSM-5 criteria confirmed by consultant psychiatrists or psychiatric medical officers using structured clinical interviews and medical records” but the specific instrument (e.g., SCID-5, MINI) is not named. Please specify the instrument(s), training, version, languages, and whether inter-rater calibration was conducted. | Thank you for this valuable methodological clarification. We agree that the specification of the diagnostic instrument and procedures is essential for transparency and reproducibility. We have now clarified that DSM-5 diagnoses were established using **routine structured clinical diagnostic interviews based on DSM-5 criteria conducted by consultant psychiatrists or trained psychiatric medical officers**, rather than a standardized research interview such as SCID-5 or MINI. These clinicians were formally trained in DSM-5 diagnostic assessment as part of their postgraduate psychiatric training and routine clinical practice. Diagnoses were further verified through **medical record review.**  The diagnostic interviews were conducted in **Sinhala and Tamil**, depending on participant preference. As the diagnosis was made as part of routine clinical care prior to recruitment, **formal inter-rater calibration between psychiatrists was not conducted** for the purpose of this study. |  |
| Severity scoring: DSM-5 SUD severity (2–3 mild, 4–5 moderate, 6–11 severe) is a categorical rubric for diagnosis, not a psychometric scale. Reporting Cronbach’s α=0.89 on DSM-5 symptom items is not appropriate (the criteria are formative/diagnostic, not reflective indicators). Remove α for DSM-5 items; if you retain reliability analyses, do so only for any new multi-item scales designed to measure a single latent construct. | This alpha was added based on the previous reviwers' comment only. Initially, this was not embedded. However, we have now removed this part. |  |
| Perceived impacts: Open-ended responses were coded thematically, but the codebook, exemplar quotes, intercoder reliability for each domain, and denominator handling (multiple responses permitted) are not reported. Provide: (i) codebook in Supplement, (ii) examples per theme, (iii) how you calculated percentages (per total N vs. per respondents endorsing any item), and (iv) κ per key code if feasible. | The code book is provided in the supplementary file 1, and example quotes are provided in Supplementary file 2  Percentages for perceived impacts were calculated using the total sample (N = 427) as the denominator. Because multiple responses per participant were permitted, the summed percentages exceed 100%.  Overall interrater reliability was measured and k-= 0.82 |  |
| Polydrug use as a confounder • With 87% reporting polydrug use, nearly all “impacts” and “associations with severity” are plausibly confounded. Current analyses do not adjust for concurrent alcohol, cannabis, heroin, or tobacco. You should: o Present stratified descriptives by polydrug vs. methamphetamine-only. o Include polydrug use indicators in multivariable models (see below) or conduct sensitivity analyses excluding heavy polydrug users. | we have now **added a stratified descriptive analysis** comparing methamphetamine-only users (n = 55) with polydrug users (n = 372) in the Results section.  The stratified findings show meaningful differences between groups. Notably, the proportion of severe addiction was **higher among methamphetamine-only users (40.0%)** compared to polydrug users (**31.7%).** Daily methamphetamine use was also more frequent among methamphetamine-only users **(41.8%)** relative to polydrug users **(26.7%).** These results demonstrate that high severity and frequent use are not solely attributable to concurrent substance use.  We also revised the Discussion to clearly acknowledge the potential confounding effects of polydrug use and to interpret the findings in light of these stratified patterns.  Given the descriptive and exploratory objectives of the study, and because inferential modelling was not planned in the original protocol, we did not add multivariable analyses. Instead, we believe the stratified results provide a transparent and methodologically appropriate way to address the reviewer’s concern. |  |
| Statistical analysis—move beyond bivariate tests • The table “Associated factors for Methamphetamine use severity” is based on χ²/Fisher’s tests only. To support statements such as “severity was associated with…”, perform multivariable modeling: o Primary: Ordinal logistic regression (proportional odds) with severity (mild/moderate/severe) as outcome; test proportional-odds assumption (e.g., Brant test). If violated, use multinomial logistic regression. o Covariates: age, sex, education, income, living area, living arrangement, age of onset, frequency, route, accessibility, and polydrug indicators. Consider province fixed effects. o Report adjusted odds ratios with 95% CIs and model fit (pseudo-R², likelihood ratio tests). o Provide effect sizes for bivariate tests (e.g., Cramér’s V) even if you keep the table. • Multiple testing: You state no correction was applied. Either prespecify a primary model and outcomes to mitigate multiplicity, or apply FDR/Bonferroni for the large number of comparisons, and explicitly mark adjusted p-values. • Some internal inconsistencies appear (see Minor comments). Please audit all frequencies and denominators. 6. Figures & tables—clarity and reproducibility • Heatmaps are visually engaging but readers need numerical tables (counts and percentages) in the main text or Supplement for each impact domain. State clearly that items were multiple-response and give the exact denominator used for each percent. | We appreciate the value of multivariable modelling in explanatory epidemiological work. However, after careful consideration, we respectfully maintain that multivariable regression is **not appropriate, not feasible, and not aligned with the purpose, design, or approved protocol** of our study. We provide a detailed justification below, along with the substantial improvements we have incorporated based on the reviewer’s feedback.  **Our Study Objectives and Design Do Not Justify Multivariable Modelling**  Our study was explicitly designed as a descriptive and exploratory cross-sectional study, focusing on:   1. Describing patterns and severity of methamphetamine use 2. Identifying the prevalence and types of polydrug use 3. documenting perceived physical, psychological, and social impacts 4. Exploring bivariate associations between severity and selected factors   The study was not designed to test a predictive model or quantify adjusted relationships. Therefore, adding multivariable logistic or ordinal regression analyses would constitute a major analytic shift beyond our prespecified aims and could introduce inappropriate causal interpretations.  This statistical approach was included in **the study protocol approved by the Ethics Review Committee**, and deviating from it would reduce methodological transparency and violate the principle of prospectively defined analysis.  ****Our Dataset Is Not Suitable for Multivariable Modelling****  The reviewer’s proposed model includes over **10 covariates**, including several with extremely low cell counts:   - Injection route - Swallowing route - Female sex - Some income and province categories with sparse frequencies   Such sparse distributions violate assumptions of:   - logistic regression - multinomial/ordinal models - proportional-odds testing - stability of coefficient estimation   This would lead to:   - convergence failures - inflated standard errors - unstable/meaningless adjusted odds ratios - risk of overfitting (events-per-variable ratio too low)   Thus, a multivariable model would **not be statistically valid or informative** for this dataset.  Further Multivariable regression inherently suggests, causal inference, or predictive modelling.  Neither is appropriate for self-reported, cross-sectional data affected by:   - recall bias - high collinearity across behavioral exposures - extremely high prevalence of polydrug use (87%) - overlapping psychosocial determinants   Introducing adjusted models may unintentionally lead readers to infer **causal relationships**, which is not supported by this study’s design.  We respectfully clarify that multiplicity corrections (Bonferroni/FDR) are **not appropriate** in exploratory descriptive studies where the purpose is not hypothesis testing, but pattern identification.  Instead, we explicitly state:   - No correction for multiple testing was applied because the study is exploratory. - Findings are interpreted cautiously, with emphasis on pattern consistency rather than p-values.   This approach aligns with current recommendations for descriptive observational research |  |
| Table footnotes should define all categories (e.g., “Urban/Semi-urban/Rural” operationalization; “Isolation from family” vs. “lack of family closeness”).  Ensure consistent capitalization (use “methamphetamine” not capitalized unless sentence-initial). | We agree that clearer operational definitions improve the interpretability of the tables. We have now added explicit footnotes defining all relevant categories, including “Urban/Semi-urban/Rural” and the distinctions between “Isolation from family,” “Lack of family closeness,” and other family-related variables. These definitions are now provided directly beneath the relevant tables to ensure clarity for readers.  Corrected |  |
| Standardize decimal precision (e.g., one decimal place for percentages) and align counts (n, %). | Thank you for this helpful suggestion. We have now standardized decimal precision throughout the manuscript. Percentages are presented to one decimal place, and counts (n, %) are aligned consistently across all tables and text (e.g., changed from 37.24% to 37.2%). These revisions have been applied to ensure clarity and consistency as recommended. |  |
| Some narrative sections over-generalize to the population of Sri Lanka or suggest policy effects beyond the sampling frame. Re-anchor claims to treatment-seeking adults at NIMH. | Thank you for this comment. We have revised the paragraph to avoid any generalizations to the broader Sri Lankan population or implications for national policy. The revised text now focuses solely on the experiences of treatment-seeking adults at NIMH and interprets easy accessibility as relevant only to this group. We also removed prescriptive statements about regulatory or enforcement measures. |  |
| Where your findings diverge from international literature (e.g., higher income and urban residence associated with greater severity), propose contextual hypotheses and acknowledge alternative explanations (clinic catchment, enforcement patterns, access/affordability, reporting bias). | Thank you for this comment. We have revised the text to include contextual explanations for why higher income and urban residence were associated with greater severity in our sample and have acknowledged alternative explanations such as clinic catchment patterns, enforcement activity, access and affordability, and possible reporting biases. We also reworded the paragraph to avoid causal language and anchor the interpretation to treatment-seeking adults at NIMH. |  |
| You excluded “acute intoxication/withdrawal” and “cognitive impairment,” relying on clinicians’ judgment. State how capacity to consent was assessed (beyond “coherent, alert, oriented”) and whether interpreters were used for non-Sinhala/Tamil speakers. Confirm whether participation affected care in any way (it should not). Consider adding a distress protocol for participants reporting suicidal ideation. | We have now expanded the description of our consent and ethical procedures. Capacity to consent was evaluated using a structured assessment in addition to clinicians’ judgment. We added details on interpreter use for participants who did not primarily speak Sinhala or Tamil, confirmed that participation had no effect on clinical care, and described the distress protocol used for participants expressing psychological distress or suicidal ideation. |  |
| Use “methamphetamine” (lowercase) consistently; reserve “ICE” to a footnote and avoid slang in academic prose. | The term **“methamphetamine”** is used consistently throughout the manuscript. The colloquial term **“ICE”** is mentioned only once in a footnote to clarify local terminology among users. |  |
| Title is long and partially duplicated; consider: “Methamphetamine use disorder, perceived impacts, and associated factors among adults receiving care at Sri Lanka’s National Institute of Mental Health: an analytical cross-sectional study.” | Corrected |  |
| Add keywords reflecting methods (e.g., “polydrug use,” “ordinal logistic regression,” “South Asia”). | Added |  |
| Abstract • include design, setting, sample, main analysis (and specify that only bivariate tests were used if multivariable is not added), principal adjusted results (after you add models), • Methods—instrument details | Study design, sample and sampling techniques, instrument details are already there. Study setting was added. |  |
| Provide the full questionnaire (English + translated versions) as Supplementary File with source citations for borrowed items and the exact DSM-5 item prompts used. | The questionnaire used in this study will be provided by the corresponding author to interested readers upon reasonable request. |  |
| Clarify how accessibility and frequency were operationalized and whether recall periods were specified. | We clarified the frequency and accessibility in foot note.  No fixed recall window was imposed, as the study relied on participants’ typical pre-treatment usage pattern, consistent with the DSM-5 approach to evaluating substance use severity. |  |
| Results—consistency • In Table 1, present n (%) consistently for all rows. Some rows show only % in text and n in table headings. Verify “Living with whom—other” count (9) vs. severity table shows “other 0” in mild category row; check alignment. | In table O1, all n (%) are already included. No missing values  In the severity table, living with whom does not imply. |  |
| A few typographical issues: stray commas/spaces, inconsistent hyphenation (semi-urban vs semi urban), and inconsistent province capitalization. | Corrected |  |
| Figures • Ensure figure captions define color scales, denominators, and that each heatmap is reproducible from the provided counts. | Figure legends clearly indicate the variability.  Generally, in the heatmap, all colour codes do not need to be defined. Instead the standard figure legend is already there by mentioning “*with color gradients representing the percentage of perceived symptoms. Darker colors indicate a higher percentage of self-reported social impact, while lighter colors indicate a lower percentage of perceived effects.”* |  |
| Some duplicates (“World Drug Report 2020” listed twice as 1 and 3). Several references are not ideal primary sources for mechanistic claims (e.g., American Addiction Centers webpage). Replace with peer-reviewed or UNODC/WHO technical documents where possible. • Check numbering order vs. first citation order; ensure in-text numbers match. | All rechecked, and changes were done. |  |
